# Cone-beam computed tomographic analysis of apical transportation and centering ratio of ProTaper and XP-endo Shaper NiTi rotary systems in curved canals: an in vitro study

**DOI:** 10.1186/s12903-021-01617-w

**Published:** 2021-05-25

**Authors:** Hamed Karkehabadi, Zeinab Siahvashi, Abbas Shokri, Nasrin Haji Hasani

**Affiliations:** 1 Department of Endodontics, Dental School, Hamadan University of Medical Sciences, Hamadan, Iran; 2 Hamadan, Iran; 3 Dental Implants Research Center, Department of Oral and Maxillofacial Radiology, Dental School, Hamadan University of Medical Sciences, Shahid Fahmideh Blvd, 6516647447 Hamadan, Iran; 4 PhD of Statistics, Tabriz, Iran

**Keywords:** Cone-beam computed tomography, Root canal preparation, Centering ratio, Rotary file

## Abstract

**Background:**

Cleaning and shaping of the root canal system is an important step of endodontic treatment. Canal transportation is a common procedural error in preparation of curved canals. This study aimed to compare the canal transportation and centering ratio of two rotary files in curved canals using cone-beam computed tomography (CBCT).

**Methods:**

Forty-four extracted human mandibular first molars with mature apices and 10° to 30° apical curvature were selected. The samples were randomly divided into two groups (n = 22) with similar curvature. The canals were prepared with ProTaper and XP-endo Shaper file systems according to the manufacturers’ instructions. The CBCT images were obtained using Cranex 3D CBCT scanner before and after root canal preparation, and canal transportation and centering ratio of the files at 3, 4 and 5 mm levels from the apex were calculated. Data were compared between the two groups using independent t-test at 0.05 level of significance.

**Results:**

The ProTaper Universal caused greater canal transportation and had lower centering ratio than XP-endo Shaper in both mesiodistal and buccolingual directions at all levels from the apex. The difference between the two groups regarding canal transportation was significant at all levels from the apex in buccolingual direction (*P* < 0.05) except for 3 mm from the apex (*P* > 0.05). The difference between the two groups regarding centering ratio was not significant (*P* > 0.05) in mesiodistal direction at all levels except for 4 mm from the apex (*P* < 0.05).

**Conclusion:**

The ProTaper Universal causes greater canal transportation in both buccolingual and mesiodistal directions than XP-endo Shaper.

## Background

Root canal cleaning and shaping is an important step of endodontic treatment [1]. Root canal preparation with hand and rotary files can cause procedural errors such as canal transportation, ledge formation, and zipping [2]. Root canal preparation of curved canals is more difficult, and the file has a higher tendency to deviate from the original canal path. Canal transportation is a common procedural error that occurs during preparation of curved canals. As the result of canal transportation in the apical third, root canal cleaning cannot be adequately performed, debris and microorganisms remain in the root canal system, and impair the integrity of root filling [3]. Evidences show that rotary nickel titanium (NiTi) files better maintain the original canal path compared with stainless steel files. In the past decades, different rotary systems have been introduced on the market with unique designs in terms of tip, taper, pitch, rake, and helical angles [2].

ProTaper Universal is the gold standard for the purpose of comparison with new rotary files [4]. Its convex cross-sectional design with a small U-shaped groove in each triangular convex blade has further improved its flexibility and decreased the incidence of apical transportation [2]. Each file pack includes three shaping instruments namely SX, S1 and S2, and three finishing instruments namely F1, F2, and F3. The files have colored rings on their shanks, which are used for identification of instrument and application according to the manufacturer’s instructions in agreement with the International Organization for Standardization [5].

The novel rotary systems have revolutionized endodontics by using smaller number of files [6]. Recently, XP-endo Shaper system was introduced, which includes a #30 file with 0.01 taper made from NiTi MaxWire alloy. The MaxWire alloy enables the instrument to undergo transformation when exposed to heat. It takes its predetermined shape when exposed to body temperature in the root canal system. Due to the use of MaxWire alloy, the XP-endo Shaper files are in the martensitic phase at 20 °C, which transforms to austenitic phase when exposed to body temperature in the root canal system [7].

According to the manufacturer’s catalogue, the tip of the XP-endo Shaper file has 6 cutting blades, which enable the file to start shaping the canal after making a glide path with ISO 15 tip size and gradually increase its efficacy to reach ISO 30 [8]. The XP-endo Shaper can easily adapt to intracanal irregularities, and has optimal resistance to cyclic fatigue [9].

At present, there is no standard technique to assess the ability of instruments to remain at the center of the canal path during root canal preparation and prevent canal transportation [1]. Several methods are used for this purpose. One method is to assess the canal cross-section at different levels from the apex. This method is commonly used for direct observation of the shape and position of the canal. However, in this method, the original path of the canal prior to instrumentation cannot be preserved. The second method for this purpose is the Bramante method, in which, the canals are sectioned prior to instrumentation and then the segments are reassembled and sectioned again to assess the changes after instrumentation [10]. The third method is the superimposition of radiographs. In this method, the pre- and post-instrumentation radiographs are superimposed to determine the changes in the longitudinal shape of the canal two-dimensionally. Although this method is easy and affordable, it has limitations due to the two-dimensional nature of images [11].

Cone-beam computed tomography (CBCT) is a valuable non-invasive 3D imaging modality, which can be used for the assessment of canal shape after instrumentation. It enables the assessment of changes in the volume, surface area, cross-sectional shape, and taper of the root canal system [12]. Numerous studies have used periapical radiographs to assess the anatomical structure of the teeth and to analyze the number and location of root canals [13, 14]. However, periapical radiographs provide two-dimensional images, therefore, they cannot provide adequate information about the root canal configuration. Since the ProTaper and XP-endo Shaper rotary systems are designed for optimal cleaning and shaping, we hypothesized that both systems would be able to effectively prepare the root canals; in other words, they can remove the pulpal tissue, intracanal microbial biofilm, and toxic byproducts and create a continuously tapered canal while maintaining the original canal geometry. This ultimately allows for the delivery of irrigating solutions and intracanal medicaments as well as the three-dimensional filling of the root canal system. Considering all the above, this study aimed to assess and compare canal transportation and centering ratio after root canal instrumentation with ProTaper and XP-endo Shaper rotary files using CBCT.

## Methods

Mandibular first molars with mature apices extracted for purposes other than this study e.g. periodontal disease and extensive caries were collected. All patients consented to the use of their extracted teeth for research purposes prior to extraction at the department of oral and maxillofacial surgery, and signed an informed consent form for this purpose.The study protocol was approved by the ethics committee of Hamadan University of Medical Sciences (IR.UMSHA.REC.1397.464).

Periapical radiographs were obtained from the teeth using MinRay intraoral digital radiography system (Soredex, Tuusula, Finland) and photostimulable phosphor plate sensor (Optime, Soredex, Tuusula, Finland). Teeth with calcification, internal or external root resorption, root fracture, severe curvature, or curves in two different directions were excluded. Eventually, 44 extracted human teeth that met the eligibility criteria were selected.

The angle of curvature was measured using the Schneider’s method [15]. The Scanora software (Soredex, Tuusula, Finland) was used in order to measure the root curvature. For this purpose, a line was drawn along the longitudinal axis of the tooth. A second line was drawn from the apical foramen to the first point of curvature. The angle formed between the two lines was measured by a caliper, and the curvature angle was determined as such. Teeth with mesial root curvature between 10° to 20° in the mesiodistal plane were enrolled and divided into two groups with similar degree of curvature.

The teeth were disinfected with 5.25% sodium hypochlorite. Access cavity was prepared using a #4 high-speed round carbide bur (Dentsply Maillefer, Ballaigues, Switzerland). A #10 K-file (Dentsply Maillefer, Ballaigues, Switzerland) was introduced into the mesiobuccal canal until the file tip was visible at the apex. Next, the working length was determined as the distance between the occlusal reference point and 1 mm shorter of the length of the #10 K-file when its tip was visible at the apex. Next, the teeth were decoronated such that the working length was standardized at 19 mm. The teeth were then mounted in acrylic resin blocks in order to enhance radiography and ensure the reproducibility of CBCT images.

All teeth were scanned prior to canal preparation using Cranex 3D CBCT system (Soredex, Tusuula, Finland) with the exposure settings of 90 kVp, 10 mA and 12 s time. Next, a 15 K-file was used for glide path preparation. All canals were prepared randomly by the same operator using an endodontic electric motor (X-Smart Plus motor; Dentsply Maillefer, Ballaigues, Switzerland) according to the manufacturer’s instructions regarding the speed and torque of the files.

In group A (n = 22), the root canals were prepared using ProTaper Universal rotary system (Dentsply Maillefer, Ballaigues, Switzerland). First, SX and then S1 and S2 files were used to flare the orifice and coronal and middle thirds of the mesiobuccal canal in order to create a straight-line access. Next, the canals were prepared by F1 and F2 files to the working length.

In group B (n = 22), XP-endo Shaper (Dentaire, La Chaux-de-Fonds, Switzerland) was used for root canal preparation. In order to simulate the body temperature, the teeth were kept in water at 37 ± 1 °C during root canal preparation [7]. The file tip was introduced into the canal and then the instrument was activated during rotation, and long and slow up-and-down movements were performed. When the file reached to the working length, up-and-down motions were repeated five times to the working length and then the file was removed from the canal while rotating.

After using each file in both systems, recapitulation was performed using a #15 K-file, and the canals were rinsed with saline. Each file was used for preparation of four canals. Next, the samples underwent CBCT with the same exposure settings as those applied prior to instrumentation.

The OnDemand 3D Dental software (Cybermed, Seoul, South Korea) was used to measure the degree of root canal transportation and centering ratio at 3, 4 and 5 mm distance from the apex of the mesiobuccal canal before and after root canal preparation. The shortest distance between the mesiobuccal canal wall and the external root surface in the mesial, distal, buccal and lingual was measured and recorded. The measurements were made on CBCT scans taken before and after instrumentation as follows [16]:

The degree of canal transportation at each level was calculated using the following formula:

$$\left( {{\text{m}}1 - {\text{m}}2} \right) - \left( {{\text{d}}1 - {\text{d}}2} \right)\,{\text{in}}\,{\text{the}}\,{\text{mesiodistal}}\,{\text{plane}}\,{\text{and}}\,\left( {{\text{b}}1 - {\text{b}}2} \right) - \left( {{\text{l}}1 - {\text{l}}2} \right)\,{\text{in}}\,{\text{the}}\,{\text{buccolingual}}\,{\text{plane}},$$

where d1 is the shortest distance between the distal margin of the root and the distal margin of the un-instrumented canal, d2 is the shortest distance between the distal margin of the root and the distal margin of the instrumented canal, m1 is the shortest distance between the mesial margin of the root and the mesial margin of the un-instrumented canal, m2 is the shortest distance between the mesial margin of the root and the mesial margin of the instrumented canal, I1 is the shortest distance between the lingual margin of the root and the lingual margin of the un-instrumented canal, I2 is the shortest distance between the lingual margin of the root and the lingual margin of the instrumented canal, b1 is the shortest distance between the buccal margin of the root and the buccal margin of the un-instrumented canal, and b2 is the shortest distance between the buccal margin of the root and the buccal margin of the instrumented canal.

The answer of 0 in the aforementioned formula indicated absence of apical transportation.

The canal centering ratio was calculated at each level using the following formula:

$$\begin{aligned} & \left( {{\text{m}}1 - {\text{m}}2} \right)/\left( {{\text{d}}1 - {\text{d}}2} \right){\mkern 1mu} {\text{or}}{\mkern 1mu} \left( {{\text{d}}1 - {\text{d}}2} \right)/\left( {{\text{m}}1 - {\text{m}}2} \right){\mkern 1mu} {\text{in}}{\mkern 1mu} {\text{the}}{\mkern 1mu} {\text{mesiodistal}}{\mkern 1mu} {\text{plane}}\,{\text{and}} \\ & \quad \left( {{\text{b}}1 - {\text{b}}2} \right)/\left( {{\text{l}}1 - {\text{l}}2} \right){\mkern 1mu} {\text{or}}{\mkern 1mu} \left( {{\text{l}}1 - {\text{l}}2} \right)/\left( {{\text{b}}1 - {\text{b}}2} \right){\mkern 1mu} {\text{in}}{\mkern 1mu} {\text{the}}{\mkern 1mu} {\text{buccolingual}}{\mkern 1mu} {\text{plane}}. \\ \end{aligned}$$

**Fig. 1:**
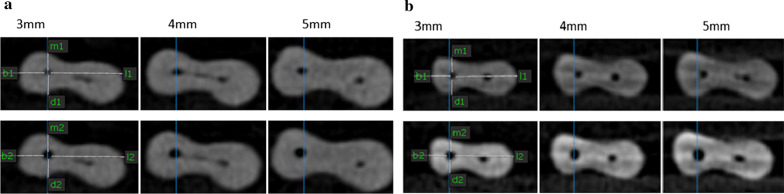
(**a**). CBCT scans before and after instrumentation of mesiobuccal canal by XP-endo Shaper at 3, 4 and 5 mm from the apex. (**b**) CBCT scans before and after instrumentation of mesiobuccal canal by ProTaper Universal at 3, 4 and 5 mm from the apex

In this formula, smaller values were placed in the numerator and the answer of 1 indicated excellent centering ability (Fig. 1a, b).

The images were assessed twice by an expert oral and maxillofacial radiologist and a dentist separately with a 2-week interval. Both observers were blinded to the group allocation of samples. They were both allowed to adjust the brightness, contrast, and magnification of images to obtain the best visual results. The mean and standard deviation values were calculated for both groups. The Kolmogorov–Smirnov two-sample test was applied to analyze the normal distribution of data. Since the data were normally distributed, independent t-test was used to compare the two groups. *P* < 0.05 was considered statistically significant.

## Results

**Fig. 2:**
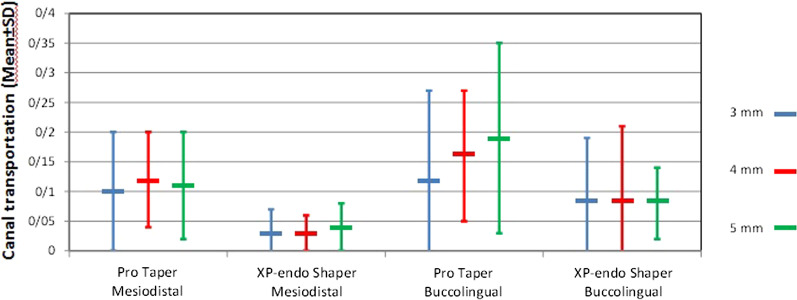
Canal transportation (Mean) at 3, 4 and 5 mm from the apex in ProTaper and XP-endo Shaper groups

In this study, in order to assess the intra- and inter-observer reliability, the intra-class correlation coefficient was calculated. The results showed inter- and intra-observer agreements > 0.87. Table 1 shows the mean mesiodistal and buccolingual transportation in the two groups. As shown, ProTaper rotary system caused greater canal transportation in mesiodistal and buccolingual directions compared with XP-endo Shaper. The difference in this respect was significant between the two systems in the mesiodistal direction at 3, 4 and 5 mm levels from the apex and in buccolingual direction at 4 and 5 mm levels from the apex (*P* < 0.05). In the buccolingual direction, the difference between the two groups was not significant at 3 mm from the apex (*P* > 0.05, Fig. 2). Canal transportation in buccolingual direction was greater than that in mesiodistal direction in both rotary systems.

**Table 1:** Mean (± standard deviation) canal transportation in the mesiodistal and buccolingual directions in the two groups

Direction	Distance from working length	3 mm	4 mm	5 mm
	Transportation	Mean(SD)		
Mesiodistal	ProTaper	0.10(0.10)	0.12(0.08)	0.11(0.09)
	XP-endo Shaper	0.03(0.04)	0.03(0.03)	0.04(0.04)
	*P* value	0.015	00	0.004
Buccolingual	ProTaper	0.12(0.15)	0.16(0.11)	0.19(0.16)
	XP-endo Shaper	0.08(0.11)	0.08(0.13)	0.08(0.06)
	*P* value	0.324	0.037	0.004

**Fig. 3:**
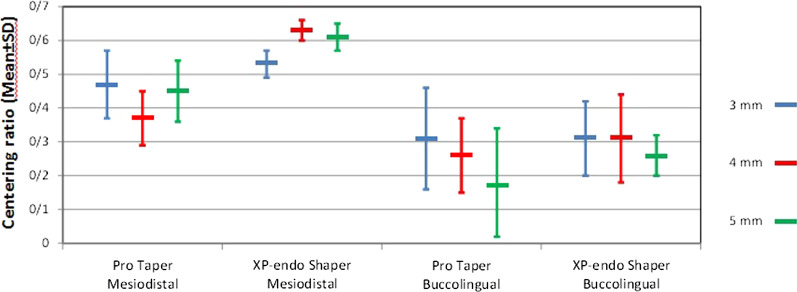
Centering ratio (Mean) at 3, 4 and 5 mm from the apex in ProTaper and XP-endo Shaper groups

Table 2 shows the mean centering ratio in mesiodistal and buccolingual directions in the two groups. As shown, the centering ratio of XP-endo Shaper in both directions was higher than that of ProTaper. In the mesiodistal direction, a significant difference was noted in this respect between the two groups at 4 mm from the apex (*P* < 0.05). However, the difference between the two groups was not significant at 3, 4 or 5 mm from the apex in the buccolingual direction, and at 3 and 5 mm from the apex in the mesiodistal direction (*P* > 0.05, Fig. 3). The centering ratio in mesiodistal direction was greater than that in buccolingual direction in both rotary systems.

**Table 2:** Mean (± standard deviation) centering ratio in the mesiodistal and buccolingual directions in the two groups

Direction	Distancefrom working length	3 mm	4 mm	5 mm
	Centering ratio	Mean(SD)		
Mesiodistal	ProTaper	0.47(0.30)	0.37(0.28)	0.45(0.28)
	XP-endo Shaper	0.53(0.33)	0.63(0.26)	0.61(0.26)
	*P* value	0.521	0.003	0.061
Buccolingual	ProTaper	0.31(0.36)	0.26(0.32)	0.18(0.25)
	XP-endo Shaper	0.31(0.38)	0.31(0.37)	0.26(0.30)
	*P* value	0.977	0.609	0.384

## Discussion

One major goal of root canal cleaning is to achieve a conical shape from the apex towards the coronal region, and maintain the original canal path [17]. As reported by Weine, when canal transportation occurs, it would be impossible to regain the original shape of the canal especially in curved canals [18]. Assessment of canal transportation and centering ratio is effective to analyze the quality of root canal preparation by different instruments and techniques [19]. According to the American Association of Endodontists, canal transportation is defined as removal of dentin from the root canal walls at the external border of the curvature in the apical half of the canals due to contact of the files since the files tend to straighten up in curved canals [20]. The centering ratio is the ability of an instrument to remain at the center of the canal path [16]. In the present study, we chose the mesiobuccal canal of mandibular first molars with 10° to 30° curvature because the risk of ledge formation, canal transportation, and perforation in curved canals, especially in the mesiobuccal canal of mandibular molars, is higher [21]. To date, two rotary file groups namely the single-file and multi-file rotary systems have been introduced on the market. This study aimed to compare apical transportation and centering ratio of XP-endo Shaper single-file and ProTaper Universal multi-file systems in extracted teeth using CBCT.

Apical transportation can be quantified using different techniques. However, each technique has its own limitations, and there is no gold standard for this purpose [22]. Among the available techniques, CBCT is a non-invasive technique for root canal assessment before and after instrumentation. It uses cone-shaped X-ray beams and an area detector for acquisition of a cylindrical volume of data. One major advantage of CBCT is to provide cross-sectional and 3D images with high accuracy and resolution. Also, it yields measurable and reproducible results [3, 23, 24].

Since the ProTaper and XP-endo Shaper rotary systems are designed for optimal cleaning and shaping, we hypothesized that both systems would be able to prepare the canals effectively.

In this study, CBCT was used to assess the canal transportation and centering ratio of two rotary systems. Aksoy et al. evaluated dentinal microcracks after root canal preparation with ProTaper Universal, XP-endo Shaper and Reciproc Blue using micro-computed tomography and observed that ProTaper Universal resulted in significantly higher number of microcracks [25]. In the present study, ProTaper showed higher canal transportation and lower centering ratio, which was in agreement with the results of Shah et al., [19] Prasanthi et al., [26] and Agarwal et al., [27] who compared ProTaper with LightSpeed LSX, self-adjusting file system, and OneShape and WaveOne, respectively [19, 26, 27]. In a study by Ozturk et al., [28] the percentage of apical transportation was 2% to 4%; also, similar to our study, apical transportation by XP-endo Shaper was lower than that by ProTaper system. Wu et al. [29] stated that apical canal transportation smaller than 0.3 mm would have minimal effect on prognosis. Thus, optimal quality of images is crucial to ensure the accuracy of results.

Greater apical transportation caused by ProTaper, compared with XP-endo Shaper (*P* < 0.05), may be due to the variable taper along the cutting blade of the file, positive rake angle without radial lands, and the triangular cross-sectional design of the ProTaper rotary system [26]. Despite the fact that triangular cross-sectional design increases the flexibility of the file, it also increases the cutting efficiency, which can lead to unwanted excessive removal of dentin from the root canal wall. Moreover, it has been demonstrated that instruments with a constant taper in their apical region have higher centering ability than those with a variable and progressive taper along their cutting blade [30]. Similar results were reported by Sonntag et al., [31] and Schafer and Vlassis [32] as they concluded that ProTaper created the highest amount of canal transportation (*P* < 0.05) when compared with Mtwo, K3 and RaCe systems.

In the present study, XP-endo Shaper showed lower canal transportation and higher centering ratio, which was in agreement with the results of Poly et al., [33] who compared XP-endo Shaper and WaveOne Gold file. Hassan et al. [34] compared the canal transportation and centering ability of XP-endo Shaper, WaveOne and OneShape rotary systems using CBCT, and showed that XP-endo Shaper better preserved the original canal shape compared with the other two systems. Their results were in agreement with our findings.

The optimal results of XP-endo Shaper may be attributed to the adaptive core technology, which helps preserve the original canal anatomy and its curvature [35]. It is believed that XP-endo Shaper applies minimum tension to dentinal walls and thus, can well adapt to the root canal irregularities. It appears that XP-endo Shaper, in contrast to ProTaper, does not have the usual shape memory of metals or tendency to straighten up. This may explain its higher centering ability and may be due to the different fabrication process of its metal alloy, which obviously affects the stress–strain distribution patterns and its flexural behavior. It also confers higher flexibility to the file and decreases its tendency to straighten up in curved canals. Many studies have demonstrated that instruments with higher flexibility have higher centering ability in root canal preparation [36–38]. Apical transportation more than 0.3 mm can decrease apical seal and compromise the prognosis of treatment [29]. Although the current study showed a significant difference in canal transportation between ProTaper and XP-endo Shaper systems, canal transportation in both systems was in the range of 0.03 to 0.19 mm, which does not compromise the apical seal. Thus, it may be concluded that both rotary systems have acceptable canal transportation with regard to preservation of the apical seal.

At 4 and 5 mm levels from the apex, ProTaper system produced the highest mean transportation compared with 3 mm from the apex; while, the XP-endo Shaper showed no statistically significant difference among different root levels in this respect. On the other hand, ProTaper showed more distal canal transportation at 5 mm from the apex, and higher tendency to cause buccolingual transportation. Therefore, it is suggested not to use instruments with a taper greater than 0.06 for apical enlargement of curved canals. Sinai [39] reported that aggressive instrumentation of the cervical third of root canals may lead to strip perforation and subsequent inflammatory complications. Less transportation towards this area can be considered as a favorable feature of XP-endo Shaper.

It has been suggested that angle of curvature alone is insufficient to adequately describe the root canal curvature, with radius being a second parameter affecting the level of difficulty of root canal treatment. Furthermore, the position of the beginning of the curve, the level where the curve occurs, the shape, and the height of the canal are other important factors to consider to better understand the root canal morphology. In the Schneider's method, however, the position, height, radius, length and shape of the root cannot be examined [40].

In the present study, the ProTaper Universal rotary system showed higher canal transportation and lower centering ratio than XP-endo Shaper in buccolingual and mesiodistal directions at 3, 4 and 5 mm from the apex.

One limitation of this study was evaluation of the teeth with moderate curvature. Future studies are required on teeth with severe curvature. Also, ex vivo and in vivo studies are warranted to confirm the current results in the clinical setting.

## Conclusion

ProTaper Universal rotary system showed higher canal transportation and lower centering ratio than XP-endo Shaper in buccolingual and mesiodistal directions at 3, 4 and 5 mm from the apex. Thus, it may be concluded that XP-endo Shaper better preserves the original canal shape.

## Data Availability

The complete documentation of all patients enrolled in this study belongs to the authors, and are available only upon reasonable request.

## Abbreviations

NiTi: Nickel titanium
CBCT: Cone-beam computed tomography
